# Intratumoral pro-oxidants promote cancer immunotherapy by recruiting and reprogramming neutrophils to eliminate tumors

**DOI:** 10.1007/s00262-022-03248-8

**Published:** 2022-08-17

**Authors:** Stephen John Ralph, Maxwell J. Reynolds

**Affiliations:** 1 School of Pharmacy and Medical Sciences, Griffith University, Gold Coast campus, Southport, QLD 4222 Australia; 2 98 Alive Pty Ltd, 5 Ridgeline Court, Kuraby, Queensland 4112 Australia

**Keywords:** Pro-oxidants, N1 and N2 neutrophils, Intratumoral, Reactive oxygen species, Cancer immunotherapy

## Abstract

Neutrophils have recently gained recognition for their potential in the fight against cancer. Neutrophil plasticity between the N1 anti-tumor and N2 pro-tumor subtypes is now apparent, as is the ability to polarize these individual subtypes by interventions such as intratumoral injection of various agents including bacterial products or pro-oxidants. Metabolic responses and the production of reactive oxygen species (ROS) such as hydrogen peroxide act as potent chemoattractants and activators of N1 neutrophils that facilitates their recruitment and ensuing activation of a toxic respiratory burst in tumors. Greater understanding of the precise mechanism of N1 neutrophil activation, recruitment and regulation is now needed to fully exploit their anti-tumor potential against cancers both locally and at distant sites. This systematic review critically analyzes these new developments in cancer immunotherapy.

**Supplementary Information:**

The online version contains supplementary material available at 10.1007/s00262-022-03248-8.

## Introduction

Neutrophils are major components (60–70%) of the white blood cell population that are gaining recognition as powerful cytotoxic agents in the fight against cancer [1, 2]. However, neutrophils also have a more sinister connotation in cancer. For example, many clinical studies have indicated that the neutrophil to lymphocyte ratio (NLR) is an important prognostic biomarker for the negative impact that higher neutrophil levels can exert on breast cancer prognosis, treatment and outcome affecting patient survival times. Over 300 reports in PubMed and several systematic reviews and meta-analyses concern “neutrophil lymphocyte ratio, and breast cancer” [3–6]. More recent studies have reported a poorer prognosis and shorter survival times for breast cancer patients with higher NLRs [7–11]. The majority of these studies indicated that an NLR greater than the cutoff value of ~ 3 was associated with worse overall survival (OS) (hazard ratio (HR) 2.56, 95% confidence intervals CI = 1.96–3.35; *P* < 0.001) and disease-free survival (DFS) (HR 1.74, 95% CI = 1.47–2.07; *P* < 0.001), with a greater prognostic value for DFS in ER-negative and HER2-negative breast cancer [4]. These findings also generally apply to all cancer types [12].

The problem with most of the clinical studies has been that neutrophils are a divergent population of immune cell types and many of these cancer studies have tended to lump them together without considering the differences in the subtypes that exist within the neutrophil population. Our recent studies with murine models of breast cancer (syngeneic, spontaneously developing ductal carcinoma in situ) have shown that when an appropriate potent pro-oxidant is delivered intratumorally, the right type of anticancer neutrophils are recruited to the site of the tumor [13]. For example, we showed that breast tumors given an intratumoral injection of a pro-oxidant treatment prepared from tea tree oil (TTO) promoted neutrophil infiltration and acted as potent cytotoxic anticancer agents that considerably suppressed tumor growths, to the point of remission [13]. Our studies are not unique and many other studies specifically applying intratumoral pro-oxidant agents such as hydrogen peroxide (H2O2) to promote anticancer tumor-associated neutrophils (TANs) contributed as the major focus of this review. The more general use of antioxidant inhibitors or other drugs working systemically as cancer therapy by mechanisms promoting reactive oxygen species (ROS), in tumors is dealt with extensively elsewhere [14–17] and will not be covered here.

This qualitative systematic review was compiled according to PRISMA 2020 guidelines [18], based on Google and PubMed/Medline searches of the literature. The flow diagram for the search analysis is shown in Supplementary Figure 1. Using keywords “neutrophil, reactive oxygen species, cancer” provided 1,111 reports, whereas “neutrophil, ROS, cancer” or “neutrophil, pro-oxidant, cancer” provided a subset of 382 and 110 reports, respectively. The majority of these related to general mechanisms involved in the production of reactive oxygen species (ROS) by neutrophils or their mechanisms of cytotoxic activation and killing of cancer cells or roles in extravasation and metastasis, which is not the main focus of this review. Several reviews on the dual role of neutrophils in cancer metastasis, effects on NETosis and extravasation and interactions with other immune cell types have recently been published [19, 20], and these provide more detailed and extensive coverage of neutrophil function. Rather, the focus here is on the intratumoral administering of small molecule pro-oxidant agents to promote tumor-associated neutrophils (TANS) as an anticancer immunotherapy. Using keywords “neutrophil, intratumoral, pro-oxidant” produced only 10 reports. Systematic filtering was applied to extract from the 1,111 articles only those were the role of neutrophils in cancer concerning the effects of ROS, their recruitment and their subtype identification was described. This review was aimed at evaluating the evidence relating to the following key points that emerged based on this comprehensive coverage of the literature and critical analysis:

1. Greater understanding emerges from identifying the particular subtypes of neutrophils which exist systemically and intratumorally, particularly with respect to the role of ROS in neutrophil recruitment and targeting of cancers,
2. Defining the distinguishing characteristics and properties of the anticancer/anti-tumor (N1) versus the pro-tumoral (N2) neutrophil phenotypes improves our understanding of their importance for cancer prognosis, and
3. Evidence supporting the direct intratumoral use of potent pro-oxidative agents to increase H2O2 produced and to reprogram the intratumoral immune cell repertoire for engaging the N1 neutrophils in the fight against cancer.

While breast cancer is often used as the selected exemplary cancer type for much of the analysis, the findings discussed also relate more broadly to the whole spectrum of cancers.

### Cancer immunotherapy should be re-evaluated with greater attention given to the neutrophil subtypes existing in cancer patients

Immunotherapy with checkpoint blockade and use of monoclonal antibody inhibitors has become a highly attractive and advantageous anticancer treatment modality showing considerable benefits across a range of cancers and more recently as a basis for improving patient survival outcomes [21]. Several recent reviews have focused on the immunotherapy for breast cancer [22–25]. However, despite the significant advances being made in improving the therapy for many types of cancers, advances in the immunotherapy of breast cancers have languished. As early as 1999, immunotherapy for breast cancer was touted as offering significant promise [26], but neutrophils did not rate a mention. In 2021, a review of the current landscape for immunotherapy of breast cancer, neutrophils and their roles were again not mentioned [27]. These observations highlight a limited understanding of neutrophils and the power that can be harnessed by reprogramming and recruiting these cells to help fight against breast and other cancers.

Studies have clearly established that polymorphonuclear neutrophils (PMNs) isolated from healthy donors exhibit potent and specific cancer cell cytotoxicity [28]. Moreover, this cancer cell killing was shown to depend on H2O2 release because addition of catalase which breaks down the peroxide to water and oxygen, inhibited the neutrophil mediated killing of the cancer cells. However, PMNs isolated from non-small cell lung cancer patients showed significantly lower ROS production levels and malignant cell killing potential [28].

The role of neutrophil ROS production and its effects on tumor cell growth or death are likely to depend on the particular neutrophil phenotype predominating within the tumor microenvironment and the local ROS levels that they release. Thus, several studies have implicated neutrophil-mediated ROS production in promoting tumor colonization [29] and chemotherapy resistance [30]. However, it is clear that by using appropriate factors it is possible to enhance and promote beneficial neutrophil recruitment and activation such as IL-17 [31], bacterial-based products [32, 33] or poly I:C double-stranded RNA [34], and these neutrophils provide potent anti-tumor immunity involving greater ROS production as detailed in the following sections. Several recent reviews have been published on the dual role of neutrophils in cancer [19, 20] and the dual roles of ROS in tumor development and progression [35]. However, none of these concerned the use of small molecule pro-oxidant drugs as agents with significant implications for promoting cancer immunotherapy by elevating intratumoral H2O2, recruiting and reprogramming neutrophils to eliminate tumors. Nevertheless, some general background is required first to enable an understanding of the relationship that neutrophils contribute to cancer.

### Distinct subtypes of neutrophils exist—antitumor N1 versus pro-tumor N2

A recent and more thorough examination of the subtypes of neutrophils responsible for immunosuppression in breast cancer identified a circulating low-density neutrophil (LDN) population that was abundant in cancer patient blood samples and increased comparing between the early to advanced stages of breast cancers [36]. An increase in LDNs commonly exists in the more advanced cancer stages and these LDNs often express the immunosuppressive surface marker, PD-L1, greater expression of neutrophil activation markers, formation of neutrophil extracellular traps (a.k.a. NETosis) and the release of ROS. The LDNs from advanced stage cancer patients were negatively correlated with levels in the blood of CD8 + cytotoxic T lymphocytes but positively correlated with the immunosuppressive CCR4 + regulatory T cells (Tregs). These LDNs were practically absent from healthy donors’ blood samples but were highly prevalent in samples from breast cancer patients with metastatic disease and were also associated with poorer responses to neoadjuvant chemotherapy compared to responders. Thus, such studies support the potential for and importance of identifying more precisely the subtypes of neutrophils to provide more useful prognostic biomarkers of breast cancer and their implication for patient outcomes [36].

Some debate has arisen recently over the use of the terms LDN versus normal density neutrophils (NDN), relating to circulating neutrophil subpopulations and their interrelationship [37, 38]. LDNs are produced from healthy NDNs in vitro by activation with pro-inflammatory factors such as TNFα, LPS, fMLF or tumor-conditioned media and CXCL1 and LDNs are closely related to NDNs in terms of morphology, functional activity and surface receptor expression, except for lower NETosis by the LDNs. Interestingly, ROS production was higher in basal (unstimulated) LDNs and significantly elevated by activation with PMA [37]. The TANs can originate from both NDN and LDN, with LDN infiltrating tumors at a higher level than NDNs. Hence, the relative density of neutrophils is not adequate for their identification as antitumor or pro-tumor and a more detailed analysis is required in order to identify them as either the N1 (antitumor) versus N2 (pro-tumor) neutrophils.

The TANs have also been linked to an immunosuppressive tumor microenvironment existing within breast cancers [39] and play a role in the aggressiveness and progression of triple negative breast cancers (TNBCs) [40]. Two recent reports showed related findings after pathology examination of breast cancer tissue samples for the presence of neutrophils within the tumor microenvironment [41, 42]. These studies identified the neutrophils by their surface marker CD66b and established a negative relationship between the higher presence of intratumoral neutrophils and the poorer response rates of patients to chemotherapy, decreased overall survival (OS) and disease-free survival (DFS). Molecular biological analyses comparing the neutrophil high intratumoral samples to those with a low level did not find any distinctive mechanism based on gene expression sets that could explain the worse survival outcomes. However, these studies did not go deep enough when exploring and defining the phenotypic differences between the different subtypes of neutrophils (N1 versus N2) involved.

Studies of other animal or human cancers have identified a neutrophil plasticity with clear TAN subtypes that exist. These findings allow for a better understanding of the situation and influence that the TAN subtypes can have on the anticancer versus pro-tumor protective aspects (reviewed in [19, 20, 43]). Several more recent reports have compared differences between these TAN subtypes, facilitating their identification and highlighting their importance to cancer [2, 44–47]. Their distinct properties can be summarized in that the N1 subtype are pro-inflammatory, express CXCL10 (a.k.a. Interferon gamma-induced protein 10; IP-10), secrete cytokines including interferons (IFNs), TNFα and others as well as chemokines that attract and promote anticancer immune cell responses. By contrast, the N2 subtype are tumor promoting and immunosuppressive, producing CCL-17 to promote immune suppressing regulatory T cells, contain high levels of nitric oxide and arginase, MMPs, elastase, are angiogenic by producing VEGF and express the surface marker, PD-L1, an inhibitory immune checkpoint impairing the anticancer T cell responses [48, 49].

The N2 subtype also produce higher amounts of TGFβ, IL-8 and IL-10 [48–51]. As breast cancers progress to reach more advanced stages, they secrete higher levels of TGFβ and several chemokines that recruit the N2 subtype, which also respond to IL-10, thereby promoting tumor cell survival, metastasis and angiogenesis.

During bacterial infection of humans, it has been shown that neutrophil production of IL-10 is induced by binding of CD11b on their surfaces to the nearby Treg cells activated to express surface ICAM-1 by the bacterial endotoxin lipopolysaccharide (LPS) [50]. Exogenous treatment with IL-10 was also shown to enhance the IL-10 production by neutrophils, indicating a positive feedback loop [50]. Hence it is highly likely that similar interactions are occurring within the immunosuppressive tumor microenvironment, where Tregs are commonly associated. Similarly, in a bacterial-induced prostatitis model, androgens were shown to promote N2 neutrophils highly expressing the anti-inflammatory cytokines IL-10 and TGFβ [52]. Furthermore, estrogens have also been shown to exacerbate tumor progression by promoting a pro-tumor microenvironment and reduced LDNs in the blood [53, 54]. Thus, multiple alternatives exist as the means for generating greater numbers of pro-tumor N2 neutrophils, supporting the immunosuppressive tumor microenvironment.

### Hypoxia and acidification support the N2 neutrophil phenotype and lower ROS production favoring an immunosuppressive tumor microenvironment.

Regions within the tumor milieu become highly hypoxic as the tumors grow in size [55]. The hypoxic tumor microenvironments (TME) activate the hypoxia-inducing factor (HIF) transcriptional response, switching cell metabolism in these hypoxic regions to favor glycolysis with greater glucose consumption and production of pyruvate, lactate and extracellular acidification [56, 57]. Hypoxia and HIF’s can also induce the production of IL-8 [58, 59] and TGFβ [60] which will recruit and enhance local production of the N2 neutrophils. Consequently, the hypoxic TME induces an immunosuppressive response with production of Treg’s, MDSCs and N2 neutrophils and increased surface expression of the checkpoint markers PD-L1 and CTLA-4 (for reviews, see [61–63]). The decreased local oxygen levels (pO2) inhibit the respiration rate and hypoxia has been shown to lower neutrophil production of reactive oxygen species (ROS), inhibiting the respiratory burst due to the lack of available oxygen [64]. In addition, TME acidification by lactate will inhibit the ROS release and ROS-dependent NETosis [65, 66], helping to maintain an immunosuppressive TME [67]. The overall effect is to stabilize the N2 neutrophils while enhancing low level release of neutrophil elastase (ELANE), myeloperoxidase (MPO), lactoferrin, matrix metalloproteinase MMP-8, and MMP-9 when compared with normoxia, representing increased secretion of the azurophilic and gelatinase containing specific granules (reviewed in [68]).

### Polarizing neutrophils into the N1 subtype to promote anticancer immunotherapy

The polarization of TANS across two extremes was first described by Fridlender based on studies of murine tumor models in which they defined neutrophils as either N1 (antitumor) or N2 (pro-tumor) phenotypes and this association also holds for human cancers (reviewed in [49]). Fridlender’s group was able to inhibit the N2 response in tumors and polarize them by shifting the balance using TGFβ blockade, which increased neutrophil-attracting chemokines, resulting in an influx of the N1 subtype CD11b( +)/Ly6G( +) TANs with hypersegmented nuclei (polymorphonuclear cells) that were more cytotoxic to tumor cells, and expressed higher levels of the pro-inflammatory cytokines [48]. Similarly, using potent immune stimulants such as Bacillus Calmette–Guerin (BCG) or beta-glucans has been found to promote reprogramming to the N1 antitumor response, capable of suppressing tumor growth in a ROS-dependent manner [69, 70]. This process required type I interferon (IFN) signaling, which also by itself will induce the N1 antitumor polarization of TANs in mice and humans [71].

Recently, the use of bacterial-based intratumoral cancer immunotherapy by applying killed mycobacteria (as complete Freund’s adjuvant) was shown to promote significant neutrophil infiltration in preclinical species as cancer models [33]. The neutrophils were essential for the anticancer effects of the bacterial induced immunotherapy and were characterized as CD11b( +)/Ly6C( +)/Ly6G9 +) TANs [33]. Survival outcomes were shown to correlate directly with the levels of the tumor infiltrating TANs. Moreover, Gr-1( +) depletion with an anti-Gr-1 antibody removed the tumor infiltrating TANS, significantly lowering cancer survival outcomes in the animal models tested. More importantly, the intratumoral treatment with Freund’s adjuvant also caused tumor reductions and extensive immune infiltrates across a range of human patients with different solid tumor types, supporting the use of such intratumoral treatments for cancer immunotherapy [33]. In some cases, distant metastases also shrank substantially, evidence indicating the onset of systemic anticancer immunity. These findings support a direct role for N1 TANS in the immunotherapy-mediated improvements in clinical responses.

In the Wistar rat breast cancer model with the Walker 256 tumor cells, it was shown that once tumors established (by 5 days after injecting 2 × 10^7^ cells subcutaneously), no further neutrophil infiltration into the TME occurred [72]. At this point, the circulating neutrophil phenotype also shifted such that they were no longer able to recognize and attack the tumor, allowing tumor growth. However, intratumoral injection of LPS altered the neutrophil population, promoting migration and activation resulting in complete tumor regression.

Interventions polarizing the human neutrophils toward either the N1 or N2 phenotypes have been studied in vitro [51]. The N2 subtype was induced from peripheral blood isolated granulocytes using factors TGFβ, IL-10 and G-CSF, whereas the N1 subtype was induced by a mixture of LPS, IFNγ and IFNβ. The N1 type was shown to be more highly activated, expressing higher CD62L and CD11b as markers for neutrophil degranulation, and greater secretion of MPO, ROS, TNF and IP-10. The N2 neutrophils produced significantly more IL-8 and were shown to be less effective at killing leishmania [51]. Similar findings were reported when transcriptomic profiles and functional differences between the N1 and N2 neutrophils were examined [73]. Compared to N2, the pro-inflammatory N1 neutrophils exhibited: i) higher levels of ROS and oxidative burst, ii) increased activity of MPO and MMP-9 and iii) enhanced chemotactic responses. N1 neutrophils were characterized by elevated expression of NADPH oxidase (NOX) subunits, as well as activation of the signaling molecules ERK and the p65 subunit of NF-kB. Moreover, the alarmin S100A9 promoted chemotactic and enzymatic activity of N1 neutrophils. Recently, cancer immunotherapy using intratumoral injection with the Toll-like receptor 3 (TLR3) agonist, poly I:C double-stranded RNA, was shown to induce an IFNγ-gene expression signature and converted the B16 melanoma immunosuppressive TME to a pro-inflammatory phenotype including greater levels of S100A9 positive TANs [74], indicative of N1 neutrophil infiltration.

To recap, the shift in cytokine and chemokine production by tumor cells as they progress to more advanced stages will contribute to the imbalance in the NLR and the immunosuppressive responses detected, particularly in the more advanced stages of cancer. Other changes in the developing TME such as hypoxia, lactate production and modified cytokine production also help to establish immunosuppression and evasion favoring more aggressive tumor progression, particularly given that high TGFβ production by advanced stage breast cancer cells is strongly immunosuppressive [75, 76] and will promote the N2 neutrophils. Moreover, the decreased oxygen maintains low levels of ROS production in the TME, supporting this immunosuppressive state.

### N1 Neutrophils are major producers of ROS and respond to ROS

Fundamental to the action of neutrophils during an immune response is the respiratory burst (or oxidative burst), involving the rapid release of high levels of ROS including superoxide anion (O2^−^) and H2O2. This ROS release can proceed as an explosive discharge upon neutrophil activation and degranulation, killing any nearby cells targeted by the ROS based suicide bomb [77, 78]. Integral to the activation of the neutrophil trigger is the assembly of the superoxide producing nicotinamide adenine dinucleotide phosphate—NOX enzyme complexes, as well as production of mitochondrial superoxide shown to be required for the neutrophil process of degranulation. Thus, the respiratory burst generates ROS by a metabolically driven oxygen-dependent process in which subunits of membrane-bound NOX catalyze the reduction of molecular oxygen to the reactive intermediate, superoxide (for review, see [79]). The reaction product, superoxide anion, is a potent ROS that rapidly undergoes further chemical and enzymatic exchanges to produce other ROS, such as H2O2, OH^−^ or nitrogen radicals (from reaction of superoxide with nitric oxide to form peroxynitrite). NOX is a multicomponent enzyme system that becomes active when four cytosolic proteins (p47phox, p67phox, p40phox and Rac2) assemble and translocate to complex with the transmembrane subunits p22phox and gp91phox. Most (~ 60%) neutrophil NOX is found associated with specific secondary granules which can be exocytosed during neutrophil degranulation, facilitating the extracellular ROS release. Gp91phox, the catalytic subunit of the NOX, is also known as NOX2.

NADPH oxidase-derived ROS are essential for neutrophil-mediated microbial killing and innate immunity and excessive ROS production induces tissue injury and prolonged inflammatory reactions [80]. Hence, production of ROS by NOX plays a paramount role in the destruction of pathogens (and cancer cells) and this process lies at the core of the cytotoxic immune reaction. IFNγ is a potent inducer of isoforms NOX1, NOX2 and NOX4 and IFNγ-primed neutrophils release higher ROS levels including superoxide anion, H2O2 and hypochlorous acid (HOCL), as well as granule lysosomal enzymes and the pro-inflammatory cytokines TNFα and IL6 [81, 82]. The enhancing effects of IFNγ on the respiratory burst were shown to occur via up-regulation of the gp91phox (NOX2) and p47phox (a.k.a. NOX organizer or NOXO1) subunits of NOX, as measured by their mRNA levels [81]. IFNα alone also primes neutrophil ROS production and maintains the transient priming effect of TNFα for several hours: It also down-regulates GM-CSF- and TNFα-activated expression of chemokine (C-X-C motif) factors, CXCL1, CXCL2, CXCL3, CXCL8, CCL3 and CCL4 characteristic of N2 neutrophils [83]. However, by contrast, IFNα also increases the expression of CXCL10 (a.k.a. IP-10) [83], a marker of N1 neutrophils. Hence, the balance between various levels of cytokines and chemokines in tumors will dictate the subtype of neutrophils they contain.

Activated neutrophils also have larger azurophilic granules called the primary granules first formed at the promyelocyte stage of differentiation, containing myeloperoxidase (MPO), lysozyme (muramidase), defensins, bacterial permeability inducer, acid phosphatase, β-glucuronidase, α-mannosidase, elastase, cathepsins B, D and G, and proteinase 3 [84]. Human but not murine neutrophils can release catalytically active ELANE that kills many cancer cell types while sparing non-cancer cells by releasing the intracellular CD95 death domain [85]. Myeloperoxidase converts chloride and H2O2 to hypochlorous acids (HOCl) that are lethally cytotoxic to cancer cells. In this manner, it is clear that neutrophils can kill the tumor cells through ROS-mediated mechanisms [86]. Mitochondrial-produced ROS (mtROS) are required in the chemoattractant-induced oxidative burst and degranulation of human neutrophils, as shown in vitro [87].

Cell-targeted ROS enhancement can also be achieved by using ROS amplifying ligands that bind to the formyl peptide receptors (FPRs) expressed on neutrophils [88]. A range of compounds can amplify the availability of ROS in neutrophils both in vitro and in vivo [88], many of these activating the NOX complex via binding to the FPRs. For example, bacterial-derived formylated peptides act as pathogen-associated molecular patterns (PAMPs) and mitochondrial-formylated peptides associated with cellular damage are considered to be danger signals acting as damage-associated molecular patterns (DAMPs) [89] triggering pro-inflammatory responses [90]. In general, bacterial derived or the mitochondrial formylated peptides from damaged cells are also danger signals activating a pro-inflammatory cell response by predominantly binding through FPR1, whereas Annexin A1 and Lipoxin A4 are known anti-inflammatory ligands of FPR2.

FPR2 can also trigger a pro-inflammatory pathway and the switch between FPR2-mediated pro- and anti-inflammatory cell responses depends on conformational changes of the receptor upon ligand binding [91]. Serum amyloid A proteins (SAAs) are acute-phase reactants secreted during responses to infection or injury and are elevated in the plasma and tumors of cancer patients [92]. SAAs can preferentially bind to FPR2 to promote IL-8 production by neutrophils [93] and SAAs act as a chemotactic signal enhancing neutrophil recruitment [94]. The exact relationship between FPR1 and 2 and the different subtypes of N1 versus N2 neutrophils has not yet been delineated. Nevertheless, activation of the FPRs (FPR1 and 2) by agonist ligands released at sites of tissue damage elicits signaling cascades that will result in neutrophil migration, activation and ROS production. Future studies will be required to determine the optimal agents for use as agonists acting via FPR1 or 2 to promote the N1 anticancer neutrophils.

### H2O2 is a ROS trigger for N1 neutrophil recruitment to target cancer

The action of the pro-oxidant induced ROS as a triggering mechanism is most likely responsible for the activated neutrophil recruitment and response that was obtained by the potent pro-oxidant tea tree oil (TTO) preparation when given as intratumoral injections into breast tumors [13]. Consequently, the number of neutrophils infiltrating into the tumors greatly increased by up to tenfold. Although they were phenotypically characterized as LDNs based on the purification process used [36, 95], the purified and enriched populations of LDNs showed potent cytotoxic anticancer cell activity when co-cultured with the breast cancer cells. These LDNs isolated from the TTO-treated tumors were identified via their surface marker expression to be CD11b + , Ly6G^hi^, Gr1 + N1 neutrophils and were the predominant immune cells infiltrating into the treated breast tumors. Furthermore, analysis of tumor sections showed marked and extensive increased presence of Gr-1 + immunostaining adjacent to TUNEL + regions indicative of cell death in the treated but not in the control untreated tumors [13].

Studies have shown that isolated populations of human peripheral blood neutrophils have a considerable capacity to absorb and utilize extracellular ROS, and remain intact when exposed over large ranges of H2O2 concentrations (10^−8^–10^−3^ M) for up to 60 min in culture [96]. It is not until the concentration of H2O2 reaches above the 10^−3^ M concentration before the neutrophil cell viability becomes compromised with release of lactate dehydrogenase (LDH). Moreover, the pre-incubation of the neutrophils at the lower range of H2O2 promoted the subsequent myeloperoxidase activity of the neutrophils to produce greater ROS levels in response to latex bead-activated phagocytosis [96].

**Fig. 1:**
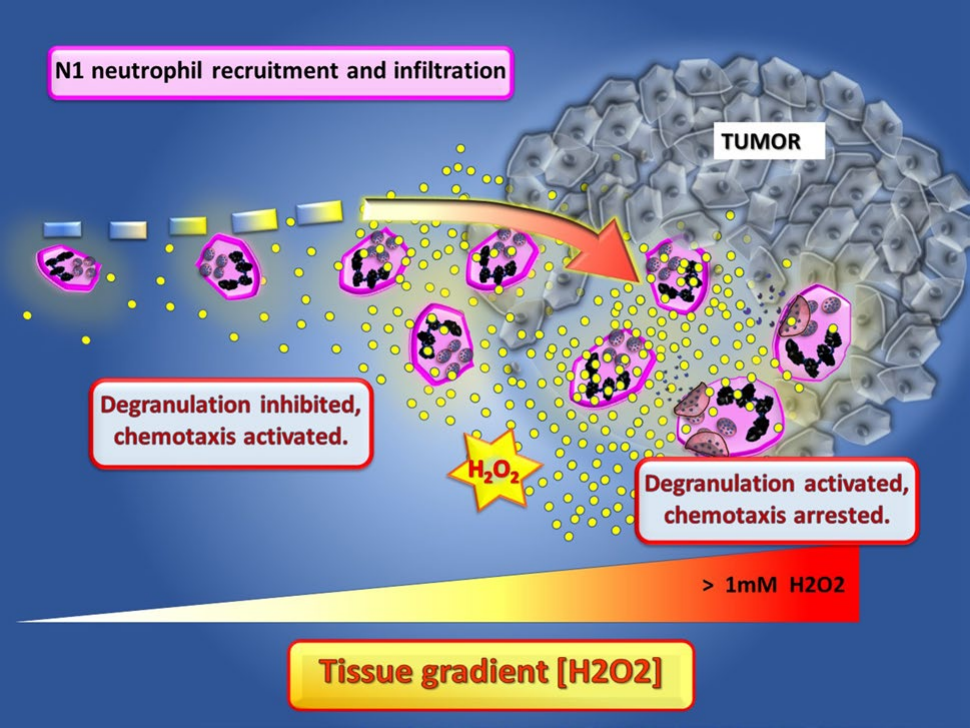
The role of H2O2 gradients extending from the tumors to promote the recruitment and activation of N1 neutrophils in the tumor microenvironment. The localized elevated H2O2-induced inside tumors produce a gradient recruiting N1 neutrophils to infiltrate and kill the tumor cells

The transient receptor potential melastatin 2 (TRPM2) proteins are Ca2 + permeable ion channels that are greatly activated by H2O2 and are integral to the regulation of neutrophil function (reviewed in [97, 98]). More recent studies have shown that H2O2 is a powerful chemoattractant for neutrophils operating both in vivo and in vitro in a manner dependent on the TRPM2 ion channel, temperature and H2O2 concentration gradient [99]. Recruitment of neutrophils was shown to remain significantly elevated even at H2O2 concentrations between 10^−8^ and 10^−5^ M but became inhibited at 10^−4^ M. It was proposed that at the higher levels, overactivation of TRPM2 floods the cell with Ca2 + , halting neutrophil cell movement [99]. This could explain the arrest of neutrophil migration once inside tumors treated with potent pro-oxidants such as the TTO preparation [13]. Similar observations have been reported relating to tissue gradients of H2O2 emanating from the site of wound healing which promotes leukocyte recruitment and migration including neutrophil and macrophages in innate immunity [100, 101]. Given the importance of ROS and particularly H2O2, this provides for the possibility of taking advantage and making use of these findings to elicit an influx of anticancer N1 neutrophils into tumors. A model for the process of H2O2-induced recruitment of neutrophils into tumors is shown in Fig. 1.

H2O2 within the TME also acts as a cue determining tumor cell susceptibility to the cytotoxic actions of neutrophils because the neutrophil-secreted H2O2 induces the lethal influx of Ca2 + into breast cancer cells via the H2O2-activated TRPM2 [102, 103]. High levels of H2O2 in the 10–30 micromolar range are required to activate TRPM2 Ca2 + permeability and cell death and catalase treatment inhibited the neutrophil cytotoxicity. Low-level expression of TRPM2 on the breast cancer cells impeded the neutrophil cytotoxic effects and promoted lung metastasis [102, 103]. TRPM2 was also shown to modulate neutrophil attraction to murine breast and Lewis lung tumor cells by regulating cancer cell expression of the neutrophil chemoattractant, CXCL2 [104]. Neutrophil-mediated H2O2 has also been shown to inhibit NK cell mediated tumoricidal activity [105] such that neutrophil activation in the presence of high levels of H2O2 will predominate and override the actions of other immune cell types in the TME [106]. Other studies have also shown that tumor-associated neutrophils can suppress the NK cell activity within tumors [107].

### Intratumorally delivered pro-oxidants as agents promoting N1 neutrophil recruitment and influx into tumors to improve targeted cytotoxic cancer immunotherapy

The emerging evidence relating to the action of pro-oxidants in promoting the TAN N1 phenotype is evaluated in this section of the review. Many pro-oxidant agents, both natural and synthetic, have been extensively tested and shown to be effective anticancer therapeutics by producing excess ROS in cancer cells, activating apoptosis (for reviews, see [108–113]. However, pro-oxidants have also been shown to regulate the neutrophil phenotypes. For example, berberine or emodin maintained the differentiation of the human myeloid leukemic cell line, HL-60 in vitro to become N1 neutrophils with higher ROS levels, but caused apoptosis of the HL-60 N2 cells [114, 115]. In addition, emodin via intragastric administration decreased N2 neutrophils and increased the ROS levels in alveolar tissues of the urethane-induced lung cancer model, preventing NETosis and increased local levels of IFNγ, IL-12 while decreasing TNFα, IL-6 and TGFβ [114]. Berberine similarly promoted the neutrophil N1 phenotype enhancing the anticancer action of Doxorubicin in the urethane-induced lung cancer model, as well as in the H22 liver cancer allograft model [115]. Treatment with Ly6G antibody to inhibit neutrophils attenuated the responses to either of the two pro-oxidants berberine or emodin and supports the role of Ly6G^hi^ N1 TANS in their anticancer actions in vivo.

H2O2 and other ROS can be produced at higher levels by cancer cells and cancer-associated fibroblasts within the TME, promoting cancer cell metabolism, growth and metastasis [35, 116–118]. While it is clear that ROS can be a tumor-promoting agent facilitating oncogenesis (reviewed in [17, 35]), this requires only moderately increased ROS levels whereas excessive amounts of ROS will cause cancer cell death [118]. The increased H2O2 is likely due to the changes in the balance between the activities of the mitochondrial Mn-superoxide dismutase (MnSOD; aka SOD2) that produces H2O2 and the two H2O2 removing enzymes, catalase and glutathione peroxidase 1 (GPX1) during oncogenesis [119, 120]. SOD removes excess superoxide radicals (O_2_^.−^) by conversion into H2O2 and oxygen (O2) while GPX1 and catalase remove hydrogen peroxide, reducing it to water. MnSOD expression is commonly increased in cancers during the progression of malignancy through invasion and metastasis [119] and would help to explain the ensuing greater H2O2 levels.

The greater levels of oxidative stress and ROS in cancer cells render them more highly susceptible to the cytotoxic effects of further elevated ROS [17]. This is because compared with normal cells, cancer cells are less able to mitigate against excessive amounts of ROS that overwhelm the redox adaptations of cancer cells. Hence, cancer cells are less able than normal tissue to cope with further elevated ROS, which causes oxidative stress levels incompatible with cell survival, leading to cancer cell death [17]. Thus, H2O2 is cytotoxic and induces apoptosis in many cell types [121] but particularly cancer cells (reviewed in [122, 123]). In 2001, based on H2O2 use as a cytotoxic agent, the direct intratumoral injection of H2O2 was proposed as a treatment for tumors [124].

A range of innovative strategies using H2O2-based tumor therapeutics have been developed aimed at overcoming the hypoxic acidified TME and producing more highly reactive oxygen species (reviewed in [125]). Early on, intratumoral injection of H2O2 was applied during tumor surgery simply for hemostatic reasons to prevent blood loss [126, 127]. Probably due to delivery issues and the instability of H2O2 in solution, not until more recently have suitably stabilized formulations of slow release H2O2 been developed and tested as direct intratumoral injections for their anticancer efficacy. However, as early as 1981, H2O2 generating systems were developed and tested as SOD mimetics capable of rapidly catalyzing the dismutation of superoxide to H2O2 and oxygen as fast or faster than the SOD enzymes. For example, in one early study, the Ehrlich Ascites Carcinoma (Ehrlich cells, EAC), a spontaneous murine mammary adenocarcinoma, grown intramuscularly as solid tumors were treated with CuII(3,5-diisopropylsalicylate)2 (CuDIPS), a copper ion-acetate-based SOD mimetic agent. Every second day, tumors were injected with 0.5 mg CuDIPS repeated for 5 or 10 doses and this therapy prolonged survival and decreased metastasis [128]. In 1990, related studies using copper-di-Schiff-based agents (formed by coordination of copper involving putrescine and either pyridine-2-aldehylde [Cu(Pu)(Py)_2_] or imidazole-2-aldehyde [Cu(Pu)(Im)_2_]) as SOD mimetics with tenfold greater activity than CuDIPS were used to treat the Walker 256 rat breast carcinoma tumors [129]. The latter Cu-chelate agents were administered by injection intratumorally on days 3, 4, 6, 8 and 10 and remarkably, low doses of 50 nmol/kg CuPu(Im)2 were sufficient to cure 50% of tumor burdened rats [129]. Higher doses of 500 nmol/kg attained 75% cure rates. More importantly, the Cu-chelate intratumoral treatments rapidly elevated the PMN levels in the blood with marked signs of acute inflammation present in areas of tumor necrosis and PMNs were noted to become highly elevated in exudates from the treated tumors. The rise in PMN levels corresponded with increased release of the intracellular enzyme, CuZnSOD detected in the plasma, most likely originating from released neutrophil granules and dying tumor tissue [129].

Recently, an important study has shown that radiation exposure can elicit a neutrophil response in the lungs that enhances metastatic colonization from primary tumors located elsewhere [130]. The degranulation of PMNs in the lung tissue was shown to induce Notch expression on the lung epithelial cells promoting the formation of metastatic lung tumors. Inhibiting neutrophils either by using Ly6G antibody depletion or by inhibiting degranulation or Notch expression impeded the lung metastases. NETosis was not required. These studies used total exposure ranges of between 8 and 13 Gy to pre-irradiate the lung tissue and induce the neutrophil infiltration. The neutrophils were identified to be of the polymorphonuclear pro-inflammatory subtype but were not identified as belonging to the N1 or N2 subtype [130]. In addition, the induced Notch expression promoted a more cancer stem cell phenotype to enhance metastatic seeding. Blocking γ-secretase activity with the inhibitor N-[N-(3,5-difluorophenacetyl)-L-alanyl]-S-phenylglycine t-butyl ester (DAPT) significantly decreased lung metastasis.

While their findings add to the complexity of the roles of pro-inflammatory neutrophils in cancer metastasis, the above studies [130] did not address the role of H2O2 release. It is known that neutrophils remain intact in the presence of low amounts of H2O2 in the micromolar range, which inhibit neutrophil degranulation [96, 131], and prevents the neutrophil production of IL-8 and IL-1β [132]. However, when H2O2 reaches levels higher than 10^−3^ molar, then neutrophils undergo degranulation [96]. In addition, the release of myeloperoxidase during degranulation has been shown to delay neutrophil apoptosis, thereby prolonging the inflammatory response [133].

Several studies have shown that the direct intratumoral administration of high levels of H2O2 itself or H2O2 generating systems significantly enhances radiotherapy outcomes to improve cancer survival rates [134, 135]. Thus, in recently reported Phase I clinical trials of locally advanced breast cancer, the use of ultrasound guided intratumoral injection of 0.5% H2O2 and 0.83% sodium hyaluronate in a gel (to slow H2O2 release and promote anticancer activity), 1 h prior to radiotherapy (36–49.5 Gy dose), was well tolerated and maintained partial or complete tumor responses relative to baseline in 11 out of 12 patients [136]. A second report followed in 2021 and the procedure is now known as Kochi Oxydol Radiation Therapy for Unresectable Carcinomas II (KORTUC II), purported to be the most widely used radiosensitizer in Japan today [137]. The second study described the results from 30 patients with locally advanced or recurrent breast cancers recording a median maximum tumor shrinkage of 97.0% and 15 patients (50%) were assessed to have achieved a clinical complete response. The proportion with loco‑regional control at 1, 2 and 3 years was 100, 94.7 and 75.4%, respectively, and progression free survival after treatment at 1 and 2 years was 59.0 and 24.1%, respectively. Based on these highly significant improvements in outcomes over standard doses of radiotherapy alone and the relatively mild side effects with complete tumor shrinkages reported in 70/71 (98%) primary breast cancers up to 5 cm diameter, a Phase II RCT of 84 participants is currently underway by the Institute of Cancer Research in the UK (https://clinicaltrials.gov/ct2/show/NCT02757651).

In other studies, injecting the H2O2 generating SOD mimetic avasopasem manganese (AVA) intraperitoneally 30 min before radiation therapy (18 Gy) was also shown to significantly and synergistically enhance the resulting anticancer outcomes [135]. The combination therapy was capable of ablating tumors in different human tumor xenograft models including lung, pancreatic as well as head and neck squamous cell carcinomas. The AVA treatment caused H2O2 production in the tumor cells because combining it with buthionine sulfoximine (BSO to deplete cellular glutathione) and auranofin (Au; to inhibit cellular thioredoxin reductase) further enhanced the AVA cytotoxicity of cancer cell lines, but not that of normal human bronchial epithelial cells. In addition, the enhanced AVA cytotoxicity when combined with BSO/Au was inhibited by the thiol antioxidant, N-Acetylcysteine (NAC), demonstrating that thiol oxidation was involved. Overexpressing the H2O2 scavenging enzyme catalase in the cancer cells obviated the effects of AVA plus radiation therapy on the xenografted tumor growth implicating ROS as H2O2 in the process. Gene expression studies showed that cytokine signaling was modified in the treated tumors with increased levels of NF-kB activation, IL-6 and TNFα [135].

The effects of the pro-oxidant TTO preparation on the tumor cells promoted high localized levels of ROS production which shifts the balance in recruitment and activation of the subtype of neutrophils to N1 and away from N2. It has been shown that NOX-mediated ROS induces NETosis by oxidizing DNA and initiating DNA repair in neutrophils [138]. NETosis in cancers is proposed to promote N2 pro-tumor neutrophils [139]. Tumor secretion of cathepsin C was shown to increase murine breast to lung metastasis by regulating neutrophils to enhance their IL-1β secretion, stimulating the production of ROS, IL-6 and CCL3 [140]. Consequently, neutrophils were recruited into metastatic niches and underwent NETosis supporting growth of the metastasized breast cancer cells whereas inhibiting cathepsin C prevented lung metastasis. In this regard, neutrophil oxidative stress and ROS production in obesity linked models of breast cancer has been  associated with neutrophil-dependent loss of vascular endothelial adhesion and cancer extravasation to the lung, associated with increased NETosis [141]. Use of catalase or blocking NETosis (with the protein arginine deiminase 4 inhibitor, GSK484) impeded breast cancer cell extravasation and neutrophil presence in the lung pre-metastatic niche of the obese animals. These studies did not address either the nature or exact levels of the ROS produced within the TME, but based on the evidence presented here are expected to be relatively low. By contrast, our findings indicated very high mitochondrial ROS production in the form of mitochondrial superoxide by tumor cells treated with the TTO preparation in vitro and in vivo and it is these extreme ROS levels that polarize toward greater numbers of N1 neutrophils within the treated tumors [13].

In other studies, we showed that the TTO preparation or terpinen-4-ol or the polyphenolic compound caffeic acid phenethyl ester (CAPE) were each capable of inducing significantly greater levels of heme oxygenase-1 (HO-1) in myeloid cell lines, while severely impeding bacterial lipopolysaccharide (LPS) induced NF-kB activation [142]. Consequently, the TTO preparation dose dependently inhibited or modified the types of cytokines produced when cells were induced by treatment with LPS. In certain medical conditions, neutrophils have been identified as the main HO-1-expressing cells present in peripheral blood, and HMOX1 mRNA expression is up-regulated by heme-moieties from lysed erythrocytes. Induction of HMOX1, the gene encoding HO-1, in neutrophils potentiates their respiratory burst [143]. Hence, the higher levels of ROS induced within the TME by TTO intratumoral injection will promote increased neutrophil HO-1 expression and sensitize them into the N1 antitumor phenotype with greater respiratory burst activation, rendering the TANs more potent in their tumor cell cytotoxic activity.

Pro-oxidants induce HO-1 expression as a feedback cytoprotective response and a means of counterbalancing against the potentially damaging aspects of greater mitochondrial ROS production levels in treated cells [144]. Neutrophils utilize their respiratory burst to release high levels of ROS which activates greater killing of the cancer cells because the addition of catalase, an enzyme that neutralizes the H2O2, abrogates the neutrophil-mediated killing of cancer cells, indicating a pivotal role for H2O2 production in this process [28, 145].

## Discussion

### Redressing the balance in favor of the N1 neutrophils with pro-oxidant agents to fight cancer

Based on a summation of the evidence, in the normal situation of untreated progressive breast cancers, the neutrophil population undergoes mobilization and a shift in the balance toward greater N2 numbers, including the TANS that become positive for surface expression of the immunosuppressive marker PD-L1 and production of TGFβ, enabling the N2 neutrophil infiltration into tumors, resident in the more advanced stages of cancers, including metastatic tumors. The outcome of these changes is to promote a more immunosuppressive environment, supporting the ongoing growth and development of tumors. In fact, inflammation-induced ROS at low levels can, by itself, also be a tumor growth and metastasis stimulant [45, 146].

**Fig. 2:**
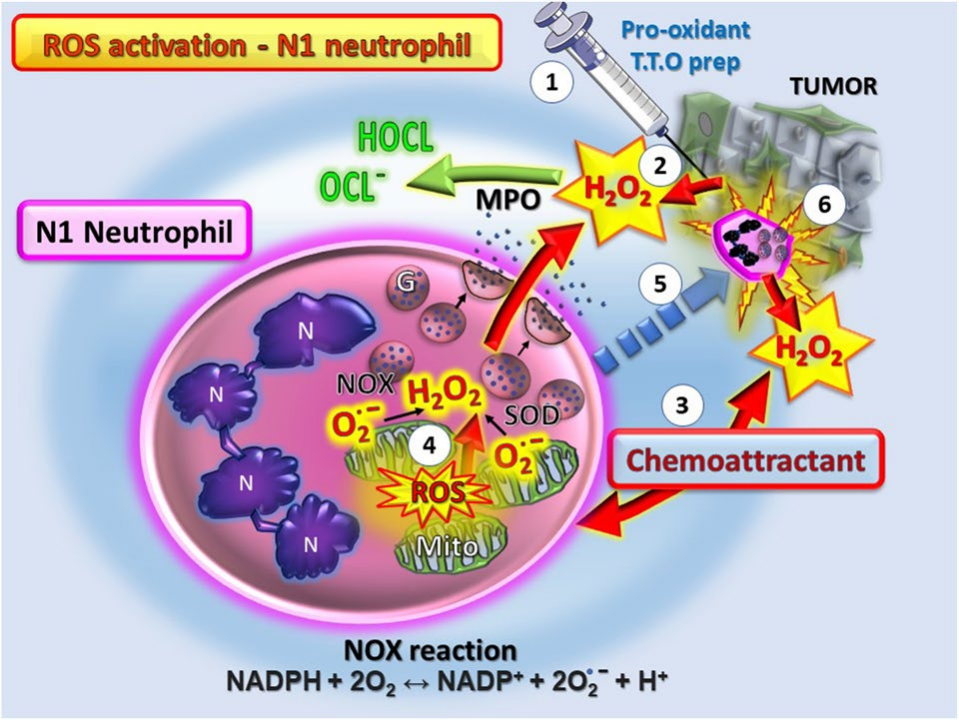
Schematic diagram showing the steps of N1 neutrophil activation occurring after intratumoral injection with pro-oxidants such as the TTO preparation. Step 1. The local action of pro-oxidant treatment is to cause severe cancer cell damage leading to step 2. Release of ROS as H2O2 and other factors that are chemoattractants (step 3) favoring recruiting N1 neutrophils to the site. Step 4. The N1 neutrophils respond to the H2O2 and increase their ROS content with heightened respiratory burst. Step 5. The overall outcome is to increase N1 tumor infiltrating neutrophils that are cytotoxic for the cancer cells (step 6) and feed-forward to induce further N1 neutrophil recruitment and eventual tumor elimination by sustained attack and oxidative stress. Abbreviations: MPO, myeloperoxidase; G, granules; Mito, mitochondria; N, polymorphic nucleus. H2O2, hydrogen peroxide; O_2_^**.**−^, superoxide; Pro-oxidant TTO prep, Tea Tree Oil preparation; HOCL/OCL^**−**^, hypochlorous acids; ROS, reactive oxygen species. The NOX reaction activated during the respiratory burst is shown beneath

However, the intratumoral injection of a potent agent such as the pro-oxidant TTO preparation causes an extremely localized mitochondrial ROS production by tumor cells, hemolysis and cancer cell cytotoxicity [13] (schematically depicted in Fig. 2). Importantly, based on the evidence presented here, the net effect is that this ensuing localized build up in ROS activates cancer cell death and causes release of pro-inflammatory factors such as TNFα, IFNs, heme and danger signals shifting the balance in favor of the anticancer immune response. Consequently, the neutrophil recruitment is altered toward the N1 phenotype of activated LDNs with heightened levels of respiratory burst and potent cytotoxic activity, leading to greater killing of the cancer cells. Sequential treatment by repeated intratumoral injection and action of pro-oxidants will continue to maintain the anticancer milieu with heightened levels of ROS and oxidative stress within the tumor, eventually causing the cancer to succumb to the multipronged attack (via the anticancer N1 neutrophil recruitment, infiltration and high local ROS production).

The question is whether the induction of a localized reaction by intratumoral injection with potent pro-oxidant agents will translate into adaptive immune responses producing a systemic immunity against other cancers residing elsewhere in the body. Thus, re-phrased, would the polarizing of the neutrophil population toward the N1 antitumor subtype carry over to impact on secondary tumor sites and metastases? In this regard, the evidence shows that neutrophils can be induced to become antigen presenting cells, expressing MHC Class II, stimulating T and B cell function and play an important regulatory role as part of not only the innate but also the adaptive immune responses (reviewed in [147]). The latter role of neutrophils in the adaptive response will be crucial for sustained long-term anticancer immunity. Moreover, neutrophils activated via immunoglobulin receptor FcγR-antibody binding have also been established as necessary and sufficient for the monoclonal antibody induced immunotherapy of tumors in mouse models of melanoma and breast cancer [148]. Hence, these studies support the essential role of neutrophils in the context of systemically enhancing immunotherapy [149] and in the current era of immune checkpoint blockade [150]—a critical area worthwhile pursing in future studies.

Additional supportive evidence is that Neutrophil-only Leukocyte Infusion Therapy (N-LIfT) is currently being tested in human trials as a cancer treatment. The N-LIfT therapy consists of N1a neutrophils as a subtype of N1 with exceptionally high anticancer selective killing across a range of cancer cell types including from pancreas, liver and lung (LIfT BioSciences). This process uses donated hematopoietic stem cells from selected non-cancer-prone individuals to prepare the N-LIfT as mass-produced cells in bioreactors for allogeneic transfer therapy. In phase I/II trials, healthy young donors provided granulocytes which were treated with G-CSF and dexamethasone and then given by allogeneic transfer to terminally ill cancer patients with advanced stage solid tumors. In a few patients, the tumors shrank with up to 80% tumor necrosis being observed [151]. Hence, these and similar studies with mouse models of cancer [152] provide solid support justifying progressing neutrophil targeting for their systemic functioning and ability to improve cancer immunotherapy.

Certain similarities exist between the divergent subtypes of neutrophils and their importance to advanced stages of cancer and to the severe pneumonia associated with increased NLR and hyperactivated neutrophils found in the acute respiratory distress syndrome (ARDS) caused by COVID-19 [78, 153]. Thus, it will be essential to consider the potential for toxic side effects or complications from over-activating neutrophils when targeting the neutrophil recruitment as cancer treatments. The induction of a cytokine storm or situation like ARDS would be highly unwarranted and undesirable and so, drug delivery via the more direct intratumoral injection for targeting potent pro-oxidants rather than a systemic treatment may be required to avoid such problems when used as an immunotherapy for cancer patients.

In conclusion, the search for more powerful pro-oxidant agents, possibly repurposing existing approved drugs with greater activity in enhancing N1 neutrophils as cytotoxic anticancer agents will lead to further improvements in the fight against cancer. Neutrophils are recognized as important immunotherapeutic targets for drug discovery [44], and their reprogramming from the pro-tumor N2 to anticancer N1 subtypes by use of potent pro-oxidants causing tumor cell cytotoxicity and release of H2O2 should help to redress the present poor status of the immunotherapy for breast cancer and other cancers. As our understanding of the mechanisms regulating neutrophil subtypes in tumors improves, this will enable more targeted interventions to promote their usefulness and exploitation when improving cancer immunotherapy in conjunction with the checkpoint inhibitors and immuno-oncology arsenal.

## Supplementary Information

Below is the link to the electronic supplementary material.

Supplementary file1 (DOCX 40 kb)

## Abbreviations

Au: Auranofin (ridaura)
AVA: Avasopasem manganese
BCG: Bacille Calmette–Guérin
BSO: Buthionine sulfoximine
CAPE: Caffeic acid phenethyl ester
DAMPS: Damage-associated molecular patterns.
DFS: Disease-free survival
ELANE: Neutrophil elastase
FPR: Formyl peptide receptor
GPX1: Glutathione peroxidase 1
HIF: Hypoxia inducing factor
IFN: Interferon
LDN: Low-density neutrophil
LPS: Lipopolysaccharide
MPO: Myeloperoxidase
mtROS: Mitochondrial ROS
NDN: Normal density neutrophil
NLR: Neutrophil to lymphocyte ratio
NOX: NADPH oxidase
OS: Overall survival
PMN: Polymorphonuclear neutrophil
ROS: Reactive oxygen species
SOD: Superoxide dismutase
TAN: Tumor-associated neutrophil
TME: Tumor microenvironment
TRPM2: Transient receptor potential melastin 2
TTO: Tea tree oil (from melaleuca alternifolia)
