# Supplementary material for: Intratumoral pro-oxidants promote cancer immunotherapy by recruiting and reprogramming neutrophils to eliminate tumors
Source: Cancer Immunol Immunother. 2022 Aug 17;72(3):527–42. doi: 10.1007/s00262-022-03248-8 (PMC9446783; doi:10.1007/s00262-022-03248-8)
Supplement: Supplementary file 1 — Supplementary file1 (DOCX 40 kb) [file 262_2022_3248_MOESM1_ESM.docx]

**Figure 1.**

**PubMED/Medline/ Google DATABASES**

Neutrophil and Cancer; n > 28,000

(in 2021, n = 2,875), exponential increase

r ~16%/yr

Neutrophil to Lymphocyte Ratio,

Breast Cancer, n = 317

Filter by neutrophil, anticancer, n= 965

Neutrophil, reactive oxygen species, Cancer, n = 1112 articles

Neutrophil, Pro-oxidant, Cancer n = 110

Neutrophil, ROS, Cancer, n = 382 articles

Filter removal relating to general mechanisms for neutrophil function and activation of respiratory burst, but not involving cancer action

Filter by neutrophil, intratumoral, pro-oxidant, n= 10

*Selection for final review & analysis, n = 151

- n = number of articles, r = exponential rate of increase per year
